# Medical Association of Georgia

**Published:** 1874-05

**Authors:** 


### Our State Societies.

### MEDICAL ASSOCIATION OF GEORGIA.

The Twenty-fifth Annual Session convened in Thomasville, on Monday, April first. Dr. G. W. Holmes, of Rome, President, in the chair. The meeting was opened with an address of welcome from Dr. T. S. Hopkins, Mayor of the city, followed by Judge J. R. Alexander, in behalf of the citizens, to which Dr. R. B. Ridley, of LaGrange, responded for the Association. Dr. W. F. Westmoreland, the President elect, was then conducted to the chair, and delivered his Annual Address. An invitation was extended by Dr. H. V. M. Miller, in behalf of the citizens of Bainbridge and the Atlantic & Gulf Railroad, for the Association to visit Bainbridge, which was accepted.

An essay on the *Medication of Nursing Children through the Mother's Milk,* by Dr. Juriah Harris, of Savannah, was presented by Dr. LeHardy; also a paper by himself, upon *the Duality of the Syphilitic Poison.* The following essays were offered: *Case of Fractured Patella with Bony Union,* by Dr. W. S. Armstrong, of Atlanta; On *Endometritis,* by Dr. J. G. Hopkins, of Thomasville: *Sympathetic Ophthalmia,* by Dr. D. W. Calhoun, of Atlanta. Dr. S. G. White, of Milledgeville, exhibited a hydrostatic injecting apparatus which he used for introducing liquids into the cavities of the body. He referred to its use for distending the whole length of the large intestine. Dr. J. T. Johnson gave the results of experiments made by Dr. Robert Battey and himself, upon the cadaver. With an ordinary Davidson's syringe they were able to throw water beyond the ileo-cæcal valve, and even on into the stomach and out at the mouth.

Essays presented: *On Dry Gangrene of both feet, result of Syphilis,* by Dr. B. S. Purse, of Savannah, accompanied by the amputated extremities: *On Compound Fracture of the Ulna with Dislocation of the Radius, and Spontaneous Amputation at the Ana-*

*tomical Neck of the Humerus*, by Dr. DeS. Ford, of Augusta. Dr. J. C. C. Blackburn reported for the committee upon *Medical Education.* Dr. T. S. Hopkins, of Thomasville, presented a paper on *the Pine Forests of South Georgia, Climate and Adaptation to the Consumptive.* Dr. R. T. Kendrick presented a paper on *Gonorrhœal Stricture, Imperforate, Remarkable Turgescence of Penis and Scrotum, Urethral Fistula.* Dr. T. F. Walker a paper *On Cases in Practice;* Also, on *Wound of Arm by Circular Saw, Grangrene, Amputation at Shoulder Joint.* Dr. Carlisle Terry, of Columbus, offered papers on *Case of Impermeable Stricture, Perineal Section, with External Urethrotomy: Case of Large Fibrous Tumor Removed from Virgin Uterus; Case of Secondary Amputation of the Thigh, one inch from the insertion of the Capsular Ligament:* Report of *fourteen cases of Compound Comminuted Fracture of the Thigh, by Gunshot Wound, with eight recoveries and but little shortening.* Dr. G. J. Grimes, of Columbus, affered a paper on *Operation for Strangulated Scrotal Hernia.* Dr. T. J. Word, of Columbus, a paper on *Woman and her Peculiarities.* Dr. Paul Faver, of Fayetteville, a paper on * * * *. Dr. T. S. Mitchell, of Harris county, a paper on *Hydrocele cured by one Tapping, the "agony of iodine not felt."* Dr. V. H. Taliaferro, of Atlanta, a paper on *Sub-involution of the Uterus.* Dr. D. W. Hammond, of Macon, a paper, *is Dysmenorrhœa induced or influenced by the size of the cervical canal.* Dr. G. W. Holmes, of Rome, a paper on * * *. Dr. DeSaussure Ford, of Augusta, a paper on * * * Dr. H. F. Campbell, of Augusta, a paper on * * *. Dr. G. F. Wirsen, of Morgan county, a paper on *Ovarian cyst; tapped in the illiac region and injected with iodine.* Also, the *Bilaterial Operation for Stone,* by Dr. F. A. Stanfor, of Columbus; Operation for *Pudendal Hernia,* by Dr. P. M. Tidwell, of Fairburn; *Ligation of Posterior Tibial Artery,* by Dr. S. G. White, of Milledgeville.

Dr. H. V. M. Miller, chairman of committee, read the following report: "The committee to whom was referred so much of the Annual Address of the President, as referred to existing laws of the State regulating the practice of medicine, ask leave to report that they have been unable to agree upon the recommendation of any action upon the subject at this time. Representing, doubtless, a respectable portion of the profession of the State, three views of the subject were advocated in the committee: Firstly, to continue in force existing laws. Secondly, to substitute for them a properly regulated system of registration in the respective counties; and, lastly, the repeal of all laws upon the subject. Without entering into argument in support of either of these propositions, the committee are unanimously of the opinion that the general assent of the profession should be secured to any law to insure its usefulness and permanency. We therefore recommend that the sentiment of the members of the Association now present be freely expressed, and the subject be remitted to the next meeting of the Association for further consideration and action. All of which is respectfully submitted. (Signed) Drs. H. V. M. MILLER, A. H. SHI, CHAS. H. HALL.

Dr. C. H. Hall read a letter from Dr. B. M. Cromwell, secretary of a meeting of physicians of Albany, held last winter, giving the substance of resolutions there passed. This meeting opposed the abolition of the State Board. Dr. S. G. White read a resolution of the "Georgia Medical Society" of Savannah, directing its delegates to "resist any effort at the repeal of the present law until a better one can be passed," and urging upon the Association the importance of some amendment to the present law to render it more effective for good. Dr. R. F. Wright, secretary of "Middle Georgia Medical Society," read a resolution of that body, requesting the Association to endeavor to procure the enactment by the Legislature, of a law confining the authority to practice medicine to the graduates of regularly chartered Medical Colleges; and requiring all physicians to register their credentials with the Ordinaries of their respective counties. A similar law to apply to the druggists, and any violation of the law to be made a penal offence. Dr. S. G. White advocated the continuance of the Board. Dr. Miller made statements showing the inefficiency of the present law, and advocated, as the first desirable step, the repeal of all laws upon the subject. Dr. T. J. Word stated that the physicians of Columbus favored the total repeal of all laws upon the subject. Drs. Ford, Thomas, and other members, briefly expressed their views upon the question. The report of the committee was adopted.

Dr. H. V. Johnson, of Hawkinsville, introduced the following resolution, which was adopted, namely: Whereas, many physicians of the regular school are in the habit of using and prescribing patent and other proprietary medicines; be it

*Resolved,* That such practice is in derogation of the honor and dignity of the profession, and should meet with the unqualified censure of this Association, and in any case, when clearly proven, shall be considered sufficient cause for expulsion from the Association.

The Annual Address was delivered by Dr. R. B. Ridley, of LaGrange. Dr. Miller called for the reading of the paper presented by Dr. T. S. Hopkins, of Thomasville, entitled "The Pine Forest of South Georgia, its Climate and Adaptability to Consumptives," which was freely discussed by Drs. Miller, White, Thomas, Ford, and Twitty. The following resolution, offer'd by Dr. Miller, was adopted:

*Resolved,* That this Association earnestly and freely endorse the opinions and statements contained in the paper just read by Dr. T. S. Hopkins; and in view of its importance to the whole country, desire to give to it the widest possible publicity.

The following resolutions were adopted, namely:

*Resolved,* That we cordially endorse the suggestions of the 24th annual meeting of the American Medical Association, to-wit: "That, in the opinion of this Association, the rank of the medical officers of the army ought to be fully equal to that of any other staff or corps, or of the medical corps of the navy; that we learn with regret that this is not the case, and that we regard with grave disapproval the odious discrimination thus made against a meritorious body of officers. That we look upon the law which prohibits promotions and appointments in the medical corps of the army as unwise and unjust, and in our opinion it ought to be forthwith repealed."

*Resolved,* That a committee be appointed to memorialize Congress on this subject, and that each member of this Association pledge himself to use his influence with the member of Congress from his district in behalf of the object of these resolutions.

A session was held upon Friday at Bainbridge, whither the Association had gone by previous invitation, which was chiefly occupied in the discussion of Malarial Hæmaturia and Yellow Fever.

The committee to whom was referred that portion of the President's address touching the call made for a convention of Confederate surgeons, offer the following:

*Resolved,* That this Association does heartily endorse the call made for a meeting in Atlanta on the 20th of May next, and that the committee be instructed to give publicity to the endorsement by this body of the call for the convention named, through the medical and other periodicals of the country.

An amendment to the Constitution was adopted, fixing the day for Annual Meeting of the Association for the third Wednesday in April.

The following officers were elected for the ensuing year, namely: President, Dr. DeSaussure Ford, of Augusta; Vice Presidents, Dr. R. L. Roddy, of Forsyth, and Dr. John M. Boring, of Atlanta; Censor, for five years, Dr. R. B. Ridley, of LaGrange. The President appointed Dr. K. P. Moore, of Knoxville, Orator of the next meeting, which is to be held in Savannah, on the third Wednesday in April, 1875.

